# DrugMGR: a deep bioactive molecule binding method to identify compounds targeting proteins

**DOI:** 10.1093/bioinformatics/btae176

**Published:** 2024-04-01

**Authors:** Xiaokun Li, Qiang Yang, Long Xu, Weihe Dong, Gongning Luo, Wei Wang, Suyu Dong, Kuanquan Wang, Ping Xuan, Xianyu Zhang, Xin Gao

**Affiliations:** School of Computer Science and Technology, Harbin Institute of Technology, Harbin 150001, China; School of Computer Science and Technology, Heilongjiang University, Harbin 150080, China; Postdoctoral Program of Heilongjiang Hengxun Technology Co., Ltd., Harbin 150090, China; School of Medicine and Health, Harbin Institute of Technology, Harbin 150000, China; School of Computer Science and Technology, Harbin Institute of Technology, Harbin 150001, China; College of Computer and Control Engineering, Northeast Forestry University, Harbin 150040, China; Computer, Electrical and Mathematical Sciences & Engineering Division, King Abdullah University of Science and Technology, KAUST, Thuwal 23955, Saudi Arabia; School of Computer Science and Technology, Harbin Institute of Technology, Shenzhen 518055, China; College of Computer and Control Engineering, Northeast Forestry University, Harbin 150040, China; School of Computer Science and Technology, Harbin Institute of Technology, Harbin 150001, China; Department of Computer Science, School of Engineering, Shantou University, Shantou 515063, China; Department of Breast Surgery, Harbin Medical University Cancer Hospital, Harbin 150081, China; Computer, Electrical and Mathematical Sciences & Engineering Division, King Abdullah University of Science and Technology, KAUST, Thuwal 23955, Saudi Arabia

## Abstract

**Motivation:**

Understanding the intermolecular interactions of ligand–target pairs is key to guiding the optimization of drug research on cancers, which can greatly mitigate overburden workloads for wet labs. Several improved computational methods have been introduced and exhibit promising performance for these identification tasks, but some pitfalls restrict their practical applications: (i) first, existing methods do not sufficiently consider how multigranular molecule representations influence interaction patterns between proteins and compounds; and (ii) second, existing methods seldom explicitly model the binding sites when an interaction occurs to enable better prediction and interpretation, which may lead to unexpected obstacles to biological researchers.

**Results:**

To address these issues, we here present DrugMGR, a deep multigranular drug representation model capable of predicting binding affinities and regions for each ligand–target pair. We conduct consistent experiments on three benchmark datasets using existing methods and introduce a new specific dataset to better validate the prediction of binding sites. For practical application, target-specific compound identification tasks are also carried out to validate the capability of real-world compound screen. Moreover, the visualization of some practical interaction scenarios provides interpretable insights from the results of the predictions. The proposed DrugMGR achieves excellent overall performance in these datasets, exhibiting its advantages and merits against state-of-the-art methods. Thus, the downstream task of DrugMGR can be fine-tuned for identifying the potential compounds that target proteins for clinical treatment.

**Availability and implementation:**

https://github.com/lixiaokun2020/DrugMGR.

## 1 Introduction

Due to the necessity of drugs in the therapy of diseases, drug development has always been a subject of great concern (Paul *et al.* 2010, Bahuguna and Rawat 2020, Theodoris *et al.* 2021). With the global outbreak of the novel coronavirus, scientists have repurposed drugs to find a treatment for the disease to shorten the research cycles and reduce costs (Ashburn and Thor 2004, Rester 2008). Conventional in vitro and in vivo experiments are reliable, but they have highly expensive and time-consuming research cycles, limiting their application (Zitnik *et al.* 2019). In contrast, identifying high-confidence ligand–target interactions through in computational methods can immensely shrink the search scope for compound candidates and provide valuable insights into the binding mechanism of protein–ligand complexes (Rifaioglu *et al.* 2019).

Over the past decade, the explosion of experimental data on bioactive molecules data has permitted the application of deep learning and artificial intelligence to the study of protein–ligand interactions. DeepDTA (Öztürk *et al.* 2018) utilized the only sequence (1D representations) for predicting the binding affinities of ligand–target pairs with two parallel convolutional neural networks (CNNs), and it exhibited promising results. Inspired by DeepDTA, Zeng *et al.* (2021) proposed a CNN-based framework with relation-aware self-attention blocks to extract the relative distance information of atoms in molecules. In addition, Abbasi *et al.* (2020) developed DeepCDA, which combined CNNs with long short-term memory (LSTM) to allow modeling for both the global and local characteristics of biological sequences. More recently, attention-based deep learning methods have been widely applied in natural language processing and computer vision fields, particularly in computational biology (Vaswani *et al.* 2017, Öztürk *et al.* 2018, Huang *et al.* 2021). Huang *et al.* (2021) mentioned a novel model named Moltrans, to predict the interaction between target–ligand pairs based on multi-head self-attention. MGPLI (Wang *et al.* 2022) also proved this perspective through treating the chemical sub-structure information as fragment-level features with transformer-CNN units and the prediction performance is highly improved. Bai *et al.* (2023) introduced an interpretable bilinear attention network framework (DrugBAN) with domain adaptation to visualize the contributions of each sub-structure to its binding regions. Drawing on the success of graph representations of molecules (Nguyen *et al.* 2021, Liu *et al.* 2022, Xuan *et al.* 2022), many graph-based methods are proposed to model the natural atom environments, allowing the domain-specific details for each target–ligand pair to be learned. GraphDTA (Nguyen *et al.* 2021) and IGT (Liu *et al.* 2022) are two masterworks that were based on graph networks for predicting binding affinities with protein–ligand complexes. Li *et al.* (2020) developed a multi-objective neural network framework, named MONN, which used a graph warp unit to extract both local and global features of molecules, significantly improving the compound-protein prediction performance. Recently, variational autoencoders (VAE) have also exhibited powerful predictive ability for the prediction of protein–ligand interaction. Li *et al.* (2022b) proposed co-regularized VAE (Co-VAE) algorithm to model the latent features of the joint distribution of triplets (ligand, target, affinity) and reconstruct the ligand and target. The predicted binding regions for the reconstructed molecules can be highlighted, which is conducive to its visualization in the prediction of protein–ligand binding regions.

While the predictive results are promising, two pitfalls remain for existing deep learning-based methods. The first is that most biological feature extraction models have inadequate ability to capture the multigranular ligand feature. The binding affinity of protein–ligand complex is essentially decided by the comprehensively natural mechanisms, e.g. the atomic environment, chemogenomic sequences, and mutual effects. However, many previous models simply represented features by separate encoders, without combinding multigranular information. Empirically, this may bring difficulties to interpret how real interaction patterns influence the protein–ligand complexes, even if the result is promising (Wang *et al.* 2022, Bai *et al.* 2023). The second flaw is that many existing methods ignore the interpretability of binding regions in model construction, which is crucial for the improvement of protein–ligand binding prediction (Lee and Nam 2022). Although a few methods inferred the binding regions via attention mechanisms with the high response, they failed, as the highlighted regions are not associated with the corresponding biological features of the target. A generalized model should be able to highlight the binding regions with high confidence for guiding researchers in locating binding sites.

To mitigate these pitfalls, we propose DrugMGR, a model based on deep multigranular representation that can predict the binding affinities and regions of ligands to protein targets. Specifically, we first use three deep modules to comprehensively encode the natural mechanisms of ligands, namely, using graph attention networks (GATs) to model atomic environments (Nguyen *et al.* 2021), CNNs to extract global chemogenomic sequences (Öztürk *et al.* 2018), and molecular transformers (MTs) to capture the mutual effects of local sub-structures (Vaswani *et al.* 2017). We also design a parallel VAE module to learn the high-level features of proteins via CNN blocks, in a probabilistic encoder, and subsequently reconstructing the target structures in a probabilistic decoder (Li *et al.* 2022b). Then, the encoded representations of ligands and proteins are fed into a pairwise interaction mapping module consisting of attention networks to learn the interaction pattern of protein–ligand complexes. The joint pairwise interaction representations are decoded by fully connected networks to predict the binding affinities of bioactive molecules. For the binding region prediction, we first highlight the binding sites of reconstructed proteins with the potential to bind with ligands as original binding areas. Subsequently, we multiply the multigranular ligand features with protein features using convolution operation. Following this, we record the convolution results as the response vector for each ligand–target pair and label the area with high values in the response vector as the visualized binding area. Finally, we utilize the two areas to guide the final predicted binding regions. Compared with DrugBAN (a binary classifier for simple interaction identification between drugs and targets), the proposed DrugMGR can further understand the comprehensive binding information (binding affinities and binding regions) of the protein–ligand complex, which plays a central role in real-world applications for bioactive molecular binding.

The main contributions of our study are summarized as follows:

- We propose a deep multigranular representation method to predict the binding affinities and regions of bioactive molecules. Through multigranular representation learning of the intricate natural mechanisms of ligands and high-level features of proteins, DrugMGR significantly outperforms the state-of-the-art methods almost over all the datasets. To the best of our knowledge, this is the first model that analyzes protein–ligand complexes using graph, convolution and attention-based information simultaneously.
- We fully design a comprehensive experiment and ablation study to establish the importance for these considerations in complex biological characteristics, analyzing the cold-start problem for binding affinity prediction.
- We introduce a new dataset, PDBbind, for further verification of the performance of the proposed model in predicting protein–ligand binding regions. Simultaneously, we visualize the attention weights for binding regions for better prediction and interpretation.
- We apply the proposed method to support triple-negative breast cancer research, identifying the potential inhibitors and chemotherapeutic agents that strongly interact with the important cancer-related protein.

## 2 Methods

### 2.1 Overview of DrugMGR

**Figure 1.**
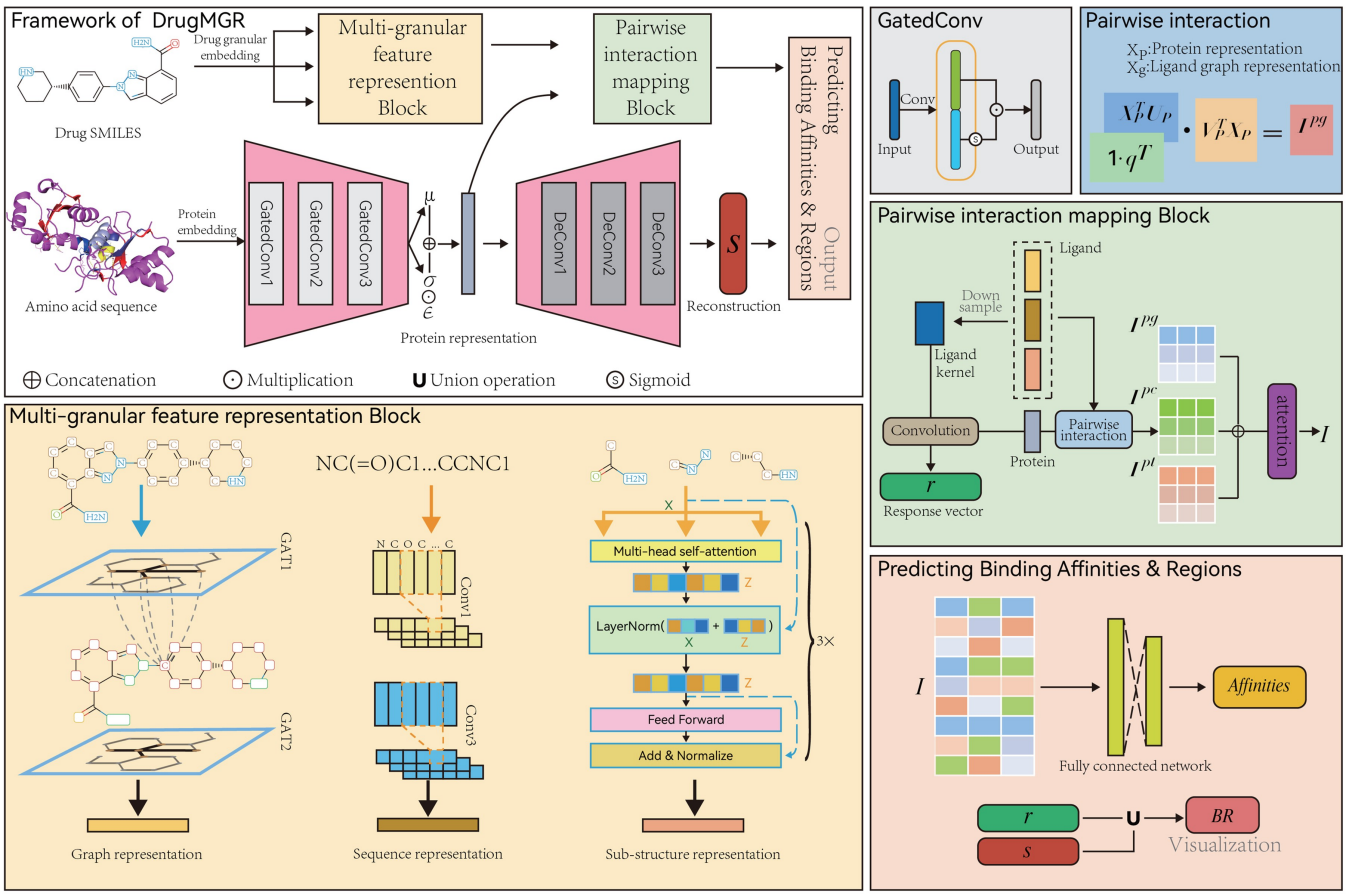
The proposed DrugMGR method for both binding affinity and region prediction. The overall scheme is presented on the left-top side. The main innovative components, such as the Multigranular feature representation block, Pairwise interaction mapping block, and Protein representation learning and reconstruction, are also demonstrated on the left-bottom side and right-hand side. Noting that, in the pairwise interaction mapping block, $I^{pg}$, $I^{pc}$ and $I^{pt}$ are the pairwise interactive features of different granular for a ligand with its protein. Specifically, $I^{pg}$ is calculated by the graph ligand feature representation (learned by graph ligand encoder) and protein feature representation, $I^{pc}$ is calculated by the sequence ligand representation (learned by CNN-based ligand encoder) and protein representation, and $I^{pt}$ is calculated by the sub-structure ligand. representation (learned by MT-based ligand encoder) and protein representation

In this study, we introduce a novel deep learning-based method called DrugMGR for predicting the binding affinity and binding region of a the ligand–protein complex. We treat the binding affinity prediction problem as a regression task in which the input is a target–ligand pair, and the output is a continuous affinity value for that pair. We represent each ligand and target as Simplified Molecular Input Line Entry System (SMILES) string and amino acid sequence, respectively. The overall pipeline for the proposed DrugMGR is illustrated in Fig. 1. Furthermore, we reconstruct the target and highlight its binding sites to identify the binding regions, improving the model performance and generalization.

### 2.2 Deep multigranular ligand representation

To represent the multigranular features of ligands, DrugMGR utilizes three parallel biological extractors. Specifically, the GATs represent each ligand as a molecular graph to extract the features of the atomic environments, and the CNNs denote the SMILES strings as a 1D sequences to learn the global information of chemogenomic sequences, and the MTs collect the most frequent consecutive sub-sequences (Sennrich *et al.* 2015) to capture the contextual sub-structure for exploring local mutual effects.

#### 2.2.1 GAT-based ligand representation learning

For the ligand compounds, we convert each SMILES string into an undirected graph G=(V,E), where node $v_{i}\in V$ is the $i_{th}$ atom in the molecule, and the edges $e_{ij}\in E$ are the chemical bonds between two atoms (Tsubaki *et al.* 2019). To align different ligands within a unified format, we set a maximum threshold $\vartheta_{g}$ for the SMILES length, those larger than $\vartheta_{g}$ are cut and those <$\vartheta_{g}$ are padded with virtual nodes zero. As a result, each ligand’s atom feature matrix is represented as $h=\{{\vec{h}}_{1},{\vec{h}}_{1},\ldots ,{\vec{h}}_{\vartheta_{g}}\},{\vec{h}}_{i}\in R^{78}$. Furthermore, a linear transformation is applied to improve the expressive power and transform ***h*** into a higher-level dense matrix $h^{\prime }=W_{g_{o}}h^{T},h^{\prime }\in R^{\vartheta_{g}\times E_{g}}$, where $W_{g_{o}}\in R^{E_{g}\times 78}$ is a learnable weight matrix and $h^{\prime }$ is the input feature.

The GAT layer takes $h^{\prime }$ as its input and calculates the attention coefficient $e_{ij}$ between node $h^{\prime }$ and its first-order neighboring nodes $h_{j}^{\prime }\in N_{i}$, connected by chemical bonds.

(1) $$\begin{array}{c} e_{ij}=\zeta ({\vec{a}}^{T}[h_{i}^{\prime }||h_{j}^{\prime }]) \end{array}$$

where $\vec{a}\in R^{2E_{g}}$ is a shared attention weight vector, ‖ and ζ are concatenation operator and nonlinear activation function, respectively. We subsequently normalize the attention coefficient $e_{ij}$ to update the weights and compute the correlation strength of the central atom and its neighboring environment.

(2) $$\begin{array}{c} {\vec{v}}_{i}^{\prime }=\sigma (\sum_{j\in N_{i}}\alpha_{ij}{\vec{h}}_{j}^{\prime }) \end{array}$$

where the attention coefficient is normalized via $\alpha_{ij}=\mathrm{softmax}(e_{ij})$. ${\vec{v}}_{i}^{\prime }\in R^{L_{g}}$ forms the output of the GAT layer and σ is a nonlinear activation function. Furthermore, we import the multi-head attention mechanism into the first GAT. A global max pooling (gmp) is followed to reduce the size and number of feature graphs to improve computing efficiency. Thus, we obtain the GAT-based ligand feature representation $X_{g}\in R^{L_{g}}$.

#### 2.2.2 CNN-based ligand representation learning

Following DeepDTA, the SMILES strings are labeled as numerical sequences using integers to digitalize the character input through scanning $∼10^{6}$ compound sequences from PubChem (Bolton *et al.* 2008) and recording 64 unique letters. Thus, a ligand SMILES {C, O, C, 1, …,), C, l} is converted into {42,48,42,35, …, 31, 42, 25}. To maintain the consistency of the graph and sequence representations, the maximum length is fixed as $\vartheta_{c}$. The ligand is then encoded as $L_{c}\in R^{\vartheta_{c}\times 64}$. Drawing on the concept of wording embedding, we utilize a learnable matrix $M_{c}\in R^{64\times E_{c}}$ to characterize the latent features of chemogenomic sequence $X_{\mathrm{seq}}=L_{c}M_{c}\in R^{\vartheta_{c}\times E_{c}}$. We then feed the sequence inputs into three convolution blocks:

(3) $$\begin{array}{c} X_{c}=FC(X_{\mathrm{seq}}+CNN_{\left(3\right)}(X_{\mathrm{seq}},W_{\mathrm{cnn}},K_{f})) \end{array}$$

where $X_{c}\in R^{L_{c}}$ is the output features of the CNN-based module, and $L_{c}$ is the output length of a CNN-based ligand feature. $W_{\mathrm{cnn}}$ and $K_{f}$ denote the numbers of filters and kernel size, respectively. FC() is a fully connected layer. $CNN_{\left(3\right)}$ includes three large-kernel 1D convolution layers, three batch normalization layers and three ReLU layers. We also adopt a residual setting (He *et al.* 2016) for the convolution blocks.

#### 2.2.3 MT-based ligand representation learning

In this part, the ligand sequences are hierarchically decomposed into consecutive sub-sequences. We first build a corpus that contains all tokenized sub-sequences via scanning massive unlabeled data over 1 870 461 ligand strings from ChEMBL (Gaulton *et al.* 2011). Following this, each ligand is encoded as a consecutive sub-structure matrix $L_{t}\in R^{\vartheta_{t}\times l}$, where $\vartheta_{t}$ is the length of the sequence, and *l* is the maximum length of the sub-structure. Before we feed it into the molecular transformer encoders, the sub-structure representation is embedded through looking up the learnable dictionary $M_{up}\in R^{E_{t}\times l}$ and then adding the position signal $M_{\mathrm{pos}}\in R^{\vartheta_{t}\times E_{t}}$.

(4) $$\begin{array}{c} X_{\mathrm{sub}}=M_{up}L_{t}+M_{\mathrm{pos}} \end{array}$$

where $X_{\mathrm{sub}}\in R^{\vartheta_{t}\times E_{t}}$ indicates the hidden features of ligand’s sub-structure. Inspired by Dong *et al.* (2023), we utilize three augmented molecular transformer encoders to extract the hidden features of local sub-structure information.

(5) $$\begin{array}{c} X_{\mathrm{att}}=\sigma (FC(\mathrm{MSA}(X_{\mathrm{sub}},H)))+X_{\mathrm{sub}} \end{array}$$

(6) $$\begin{array}{c} X_{\mathrm{mlp}}=FC(X_{\mathrm{att}})+X_{\mathrm{att}} \end{array}$$

where $X_{\mathrm{att}}\in R^{\vartheta_{t}\times E_{t}^{\prime }}$ and $X_{\mathrm{mlp}}\in R^{\vartheta_{t}\times E_{t}^{\prime }}$ are the output features for multi-head attention and fully connected layers in a transformer encoder. MSA() is a multi-head attention function and H divides the head numbers. Each transformer encoder has two pieces of layer normalizations and two residual layers. The output of the MT-based ligand features $X_{t}\in R^{L_{t}}$ is represented by a multilayer perceptron following the consecutive transformer encoders.

### 2.3 Protein representation learning and reconstruction

VAE can easily recognize the latent variable ***z*** from a given input through the combination of the techniques of deep learning and Bayesian machine learning (Li *et al.* 2022b; Xuan *et al.* 2022). Thus, we introduce a VAE module with gated convolution networks (GatedCNN) (Dauphin *et al.* 2017) to represent the high-level features of a protein in a sequence form. Notably, the VAE block for protein sequence representation is not pre-trained over any unlabeled dataset. GatedCNNs can learn features better than traditional CNNs, saving significant training resources, and can avoid problems such as gradient disappearance. More importantly, this GatedCNN-based VAE structure improves the capability of locating the real binding sites for a protein, which leads to a high promotion for binding region prediction.

Similar to the encoding operations of multigranular ligand representations, the protein sequence is encoded as feature matrix $L_{p}\in R^{\vartheta_{p}\times D_{p}}$ that contains local residue patterns, where $\vartheta_{p}$ and $D_{p}$ are the maximum lengths of a protein and hidden information of amino acid, respectively. Here, the protein sequences are considered as overlapping 3-mer amino acids (Li *et al.* 2022a), i.e. MTVKTE…DSFL→MTV, TVK, VKT, KTE, …, DSF, SFL. The VAE-based module for protein feature representation involves a decompose–reconstruction process with variational distribution through the construction of a probabilistic encoder (GatedCNNs) and a decoder (consisting of deconvolution layers). The output of the encoder and decoder can be represented as follows:

(7) $$\begin{array}{c} X_{en}=FC(\mathrm{Rep}(\mathrm{GatedCN}N_{\left(3\right)}(L_{p},W_{en},K_{en}))) \end{array}$$

(8) $$\begin{array}{c} X_{de}=FC(\mathrm{DeCN}N_{\left(3\right)}(FC(X_{en},W_{de},K_{de}))) \end{array}$$

where $X_{en}\in R^{L_{p}}$ and $X_{de}\in R^{\vartheta_{p}}$ are the high-level features and the reconstructed protein, respectively. $W_{en}$ and $K_{en}$ are the number of filters and kernel size of encoder, respectively, similar to the role of $W_{de}$ and $K_{de}$ for the decoder. $\mathrm{GatedCN}N_{\left(3\right)}$ contains three 1D-gated convolution layers, two ReLU functions and a max-pooling layer. The $W_{en}$ is doubled and tripled on in the second and the third layer, respectively. Rep() provides a reparameterization function for obtaining mean values and deviations of output features. $\mathrm{DeCN}N_{\left(3\right)}$ includes three deconvolutional layers, three ReLU functions. The VAE decoder also has two fully connected layers, where the first one transforms the protein features into new features with the correct size for the deconvolutional layers, and the other one reconstructs the protein from the output of the last deconvolutional layer.

### 2.4 Pairwise representation learning for prediction

The pairwise representation learning of protein–ligand pair plays a crucial role in DrugMGR, simultaneously extracting intractable affinity features and predicting binding regions. The input of pairwise representation learning process includes two main components: multigranular ligand features (consisting of graph atomic environments, global chemogenomic sequences, and contextual mutual sub-structures) and high-level protein features. To predict the binding regions of a protein–ligand complex, we first concatenate three ligand features as $F_{l}\in R^{L_{g}+L_{c}+L_{t}}$ and obtain the ligand kernel $K_{l}\in R^{L_{s}}$ via sampling $X_{l}$ with a linear layer. Then, the target–ligand response vector $r\in R^{\vartheta_{p}}$ is computed by filtering the protein features $F_{p}\in R^{L_{p}}$ with the ligand kernel $K_{l}$. Specifically, the $i_{th}$ element of *r* is calculated as:

(9) $$\begin{array}{c} r_{i}=\sum_{m=0}^{L_{s}}K_{l}(m)*W_{bs}F_{p}(i) \end{array}$$

where $W_{bs}\in R^{\vartheta_{p}\times L_{p}}$ is a learnable weight matrix, and * denotes the Hadamard product. In the response vector, the highest values are labeled as binding sites. We also highlight the binding sites for the reconstructed protein sequence and regard them as the original binding areas $s\in R^{\vartheta_{p}}$. Then, the predicted binding regions are obtained as BR=r∪s, where ∪ represents the public space of *r* and *s*. To capture the binding affinity features, we construct pairwise interaction matrix $I^{pg}\in R^{L_{p}\times L_{g}}$ with bilinear attention networks.

(10) $$\begin{array}{c} I^{pg}=(1\;\cdot \;q^{T})*\sigma (X_{p}^{T}U_{p})\cdot \sigma (V_{g}^{T}X_{g}) \end{array}$$

where $U_{p}\in R^{L_{p}\times C}$ and $V_{g}\in R^{L_{g}\times C}$ are learnable weight matrices for graph ligand and protein representations, $1\in R^{L_{p}}$ is one vector and $q\in R^{C}$ is a learnable weight vector. In a similar fashion, the interaction matrices $I^{pc}\in R^{L_{p}\times L_{c}}$ and $I^{pt}\in R^{L_{p}\times L_{t}}$ for sequences and mutual sub-structures with proteins are calculated. Following this, we concatenate them and use an attention mechanism (Vaswani *et al.* 2017) to enrich the complex features of the protein–ligand pairs:

(11) $$\begin{array}{c} X_{\mathrm{out}}=IW_{a}*f_{a}(I) \end{array}$$

(12) $$\begin{array}{c} f_{a}=\mathrm{Softmax}(\frac{IW_{m}}{\sqrt{L_{g}+L_{c}+L_{t}}}) \end{array}$$

(13) $$\begin{array}{c} I=\mathrm{Concat}(I^{pg}+I^{pc}+I^{pt}) \end{array}$$

where $X_{\mathrm{out}}\in R^{L_{p}\times (L_{g}+L_{c}+L_{t})}$ forms the output of the attention mechanism, and $W_{a}$ and $W_{m}$ are the parameter matrices. $f_{a}()$ is a linear normalization to improve the regions with a significant influence on the interaction pattern in the interaction matrix *I*. We sample $X_{\mathrm{out}}$ using global average pooling and linear layer to obtain the final interaction features $V_{\mathrm{out}}\in R^{L}$.

### 2.5 Training implementation

For affinity prediction, we follow GraphDTA (Nguyen *et al.* 2021) and use *Mean Square Error* (*MSE*) as the loss function, $\mathrm{MSE}=\frac{1}{n}\sum_{i=1}^{n}{\left(P_{i}-Y_{i}\right)}^{2}$, where *P* is the predicted affinities, *Y* shows the affinity labels, and *n* indicates the number of samples. For region prediction, we first gather the binding site information on proteins from the PDB dataset (Berman *et al.* 2000) and encode them as label vectors with lengths of $\vartheta_{p}\times 1$. We convert elements not in binding regions into 0 and this in binding regions into affinity values to match the interaction strength of a protein–ligand complex. However, the encoded label vector only has a small proportion of affinity values. Inspired by Feng *et al.* (2020), we adopt *Rwing* loss function for the binding region prediction training process to adress this problem. We first use the mature DrugMGR, which has been trained on the binding affinity datasets and fine-tuned it with the PDBbind dataset. Subsequently, we jointly use the *MSE* and *Rwing* as the ultimate loss, which can be represented as $\mathrm{Loss}=\psi L_{BA}+(1-\psi )L_{BR}$, where $L_{BA}$ and $L_{BR}$ indicate the loss of affinity and region prediction (i.e. *MSE* and *Rwing*), respectively, and ψ stands for a weight parameter controlling the contribution of $L_{BA}$ and $L_{BR}$ to the final prediction. According to the grid search for ψ, the value of 0.4 yields the optimal results. The hyper-parameters in our training process are carefully introduced in supplementary materials.

## 3 Experiments and results

### 3.1 Experiment setting

**Dataset:** This study evaluates DrugMGR on three widely accepted benchmarks: the Davis dataset (Davis *et al.* 2011), the KIBA dataset (Tang *et al.* 2014), and the BindingDB dataset (Gilson *et al.* 2016). Noting that, the *MSE* was used only when training the DrugMGR on these three benchmark datasets due to the absence of ground-truth labels on binding sites. In addition, we introduce a new dataset, PDBbind v.2019 (Wang *et al.* 2005), by converting the 3D information into the a sequence format to examine the predictive performance of our proposed method for binding regions. Because the same target–ligand pairs (inputs) with different or duplicate affinity values (labels) could harm the effectiveness of the training process, these abnormal candidate samples are removed from each dataset. Following Hua *et al.* (2023), we convert the $K_{d}$ value into $pK_{d}$ as affinity values: $pK_{d}=-\log(K_{d}/10^{9})$. The statistical and chemical information of datasets are presented in Supplementary Fig. S1 and Supplementary Table S2.

**Evaluation strategy:** We study the regression performance on benchmark datasets with two experimental strategies: random split setting and cold split setting. For random split setting, each dataset is randomly split into training, validation and test sets with a 4:1:1 ratio. The cold split setting, also known as a cold-start problem, includes cold-ligand and cold-target two scenarios (Nguyen *et al.* 2022). To be consistent with existing studies, the experiments are run with 10-repeated 5-fold cross-validation. Statistical significance with *P*-value < 0.05 is performed for a two-tailed test (Luo *et al.* 2022).

To evaluate the proposed model, we applied the *Concordance Index* (*CI*) (Gönen and Heller 2005), *MSE*, $r_{m}^{2}$, and *Area under the Precision-Recall Curve* (*AUPR*) metrics. The detailed information of evaluation criteria can be found in supplementary materials.

### 3.2 Performance comparison under the random split setting

Here, we compared DrugMGR with four state-of-the-art (SoTA) prediction baselines, i.e. DeepDTA, GraphDTA, MFR-DTA and DrugBAN (see supplementary materials for detailed information) under the random split setting. The evaluated results of three benchmark datasets are presented in Table 1. DrugMGR has consistently competitive performance compared with baselines in terms of *CI*, *MSE*, *AUPR*, and $r_{m}^{2}$ metrics. For the unbalanced label distribution datasets (BindingDB and Davis), the concentrated affinities may lead the model to learn the interaction features between target–ligand pairs with bias and limit its performance in the *MSE* metric. However, DrugMGR yielded a higher *CI* value and a lower *MSE* value, and its *AUPR* and $r_{m}^{2}$ metrics also outperformed the compared methods. Taking the BindingDB dataset for instance, the proposed model achieved 1.6%, 23.9%, 2.1%, and 3.5% performance gains in terms of the used metrics, respectively, over the second-best model, DrugBAN (Bai *et al.* 2023). The superiority is mainly due to our multigranular representation learning, which alleviates the problem of affinity distribution, guiding authentic interaction patterns by extracting intricate features of bioactive molecules.

**Table 1.** Performance evaluation of DrugMGR and the SoTA methods on BindingDB, Davis, and KIBA datasets.^a^

	Method	CI (std)	MSE (std)	AUPR (std)	$r_{m}^{2}$
BindingDB	DeepDTA	0.824 (0.003)	0.926 (0.031)	0.709 (0.013)	0.605 (0.024)
	GraphDTA				
	MFR-DTA	0.857 (0.004)	0.632 (0.024)	0.721 (0.007)	0.674 (0.005)
	DrugBAN	0.884 (0.004)	0.472 (0.015)	0.743 (0.004)	0.691 (0.003)
	DrugMGR	**0.898 (0.002)**	**0.359 (0.007)**	**0.759 (0.003)**	**0.715 (0.002)**
Davis	DeepDTA	0.863 (0.002)	0.194 (0.011)	0.788 (0.004)	0.673 (0.009)
	GraphDTA	0.866 (0.004)	0.179 (0.003)		0.687 (0.007)
	MFR-DTA	0.887 (0.003)	0.151 (0.004)	0.817 (0.004)	0.776 (0.005)
	DrugBAN	0.899 (0.003)	0.137 (0.002)	0.828 (0.004)	0.785 (0.003)
	DrugMGR	**0.907 (0.002)**	**0.131 (0.001)**	**0.856 (0.002)**	**0.801 (0.002)**
KIBA	DeepDTA	0.878 (0.004)	0.261 (0.007)	0.714 (0.010)	0.630 (0.017)
	GraphDTA	0.892 (0.005)	0.232 (0.004)		0.675 (0.010)
	MFR-DTA	0.895 (0.004)	0.229 (0.003)	0.741 (0.003)	0.695 (0.004)
	DrugBAN	0.906 (0.003)	0.219 (0.002)	0.753 (0.003)	0.711 (0.003)
	DrugMGR	**0.911 (0.001)**	**0.211 (0.001)**	**0.767 (0.002)**	**0.734 (0.002)**

a Bolded: best results.

When applied to the large dataset KIBA, which has a relatively normal label distribution, DrugMGR also achieved a 0.005 increase and 0.008 decline in the *CI* and *MSE* indexes, respectively, relative to the second-best model. The other metrics for the proposed method, i.e. *AUPR* and $r_{m}^{2}$, were also superior to the other SoTA methods. Due to the incredibly centralized sample labels for KIBA, the affinity trend is hard to predict, leading to an obstacle for most methods to the in terms of performance in the *CI* index. The main merit of our model is the sensitivity to distinguishing the different and common characteristics of a protein–ligand complex. Taken together, the experimental results suggest that our proposed DrugMGR almost outperforms all the compared methods for all evaluation metrics.

### 3.3 Cold-start scenario and target-specific analysis

The promising results achieved in terms of the random split setting could be due to bias and prior knowledge of learned data, not a model’s real-world performance in practical scenarios. Thus, we further conduct an experiment using a cold-start strategy to evaluate methods of alleviating the over-estimated performance under random splits due to potential data leakages. This cold split setting (cold-ligand and cold-target) promises the cold ligands or targets are not observed in the training process and thus the model cannot depend on the features of learned ligands or targets. We randomly allocate 5%/10% ligands (targets) into the validation/test set and delete all of their related targets (ligands) from the training set.

**Figure 2.**
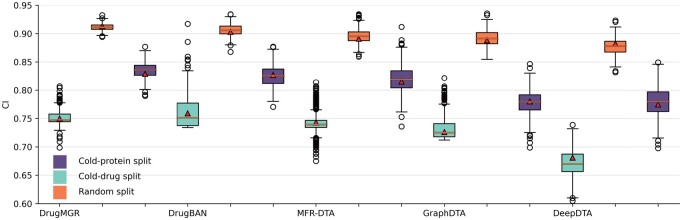
Performance comparison on the BindingDB dataset with random split and cold-start split

Table 2 records the performance in Davis and KIBA datasets under the cold-start setting. We observed that DrugMGR achieves almost the optimum performance against other SoTA methods in the cold-ligand setting. However, for the Davis benchmark, our proposed model only achieved the second-best performance in terms of the *CI* index. This could be due to two main factors. First, there were fewer target–ligand pairs in this dataset, resulting in inadequate samples to train models. Second, more diverse interaction samples in the KIBA dataset allow the model to fully learn the biological information. In the cold-target splitting, the proposed method also exhibited superiority against other predictors in both benchmark datasets. In addition, we compare the performance between random split and cold-start split settings in the BindingDB dataset. As shown in Fig. 2, in the cold-ligand split setting, all methods have a significant performance decline, while DrugMGR still outperformed all other SoTA methods. Meanwhile, in the cold-target setting, the proposed method also exhibited relatively compelling performance compared with the random split one. The main reason could be the well-designed decompose–reconstruction structure for the protein feature extractor of DrugMGR, which fully learns the latent features to improve stability.

**Table 2.** Results of cold-ligand and cold-target split analysis in Davis and KIBA datasets (C-L: cold-ligand, C-T: cold-target).^a^

	Methods	Davis		KIBA	
		CI	MSE	CI	MSE
C-L	DeepDTA	0.671	0.726	0.728	0.466
	GraphDTA	0.732	0.840	0.728	0.447
	MFR-DTA	0.754	0.719	0.745	0.402
	DrugBAN	**0.763**	0.684	0.759	0.389
	DrugMGR	0.759	**0.527**	**0.763**	**0.389**
C-T	DeepDTA	0.780	0.490	0.722	0.430
	GraphDTA	0.778	0.457	0.658	0.519
	MFR-DTA	0.819	0.421	0.739	0.422
	DrugBAN	0.826	0.411	**0.754**	0.436
	DrugMGR	**0.835**	**0.405**	0.753	**0.441**

a Bolded: best results.

To further evaluate the practical application, a target-specific compound identification task is conducted over the DUD-E (Mysinger *et al.* 2012) and the LIT-PCBA (Tran-Nguyen *et al.* 2020) datasets, we also compared DrugMGR with three such task-designed methods, i.e. MONN (Li *et al.* 2020), MFR-DTA (Hua *et al.* 2023), and IGT (Liu *et al.* 2022). The DUD-E dataset contains 22 886 active compounds and 1 411 214 decoys against 102 targets, an average of 224 ligands per target. The training-test splitting strategy is the same as MONN. The LIT-PCBA dataset is a newly built binary labeled binding activity dataset which contains 15 targets, 9780 active compounds and 407 839 inactive compounds. The train/test ratio for targets is set to 4:1 in the LIT-PCBA dataset. We evaluated all the methods under the cold-target setting. As shown in Table 3, in both DUD-E and LIT-PCBA datasets, the average *AUC* and *AUPR* score over the test proteins achieved by DrugMGR were significantly improved against other predictors. The results indicated that our proposed method is accurate and robust for identifying the target-specific compounds and can be reliable for practical application.

**Table 3.** Performance evaluation of different prediction methods on the DUD-E and LIT-PCBA datasets.^a^

Dataset	Model	AUC	AUPR
DUD-E	IGT	0.924	0.692
	MFR-DTA	0.961	0.714
	MONN	0.975	0.732
	DrugMGR	**0.982**	**0.757**
LIT-PCBA	IGT	0.897	0.311
	MFR-DTA	0.925	0.425
	MONN	**0.943**	0.487
	DrugMGR	**0.943**	**0.512**

a Bolded: best results.

In fact, the model may fail to correctly predict the binding affinities between the compounds and proteins. In some cases, a protein-targeted compound is highly similar to another compound (without interaction) in terms of chemical sequence, but their binding conformation is totally different. The slight difference in the binding core of molecules is likely to bring tremendous changes in binding conformation in spatial omics. For this reason, the model without customized representation learning modules cannot accurately learn the feature information and thus obtain a less precise result. Accordingly, we found that multigranular representation learning is of significant importance to the complex protein–ligand prediction task.

### 3.4 Ablation study

To evaluate the effectiveness of each innovative component in DrugMGR, a comprehensive ablation study is conducted. In this part, we set the DeepDTA (Öztürk *et al.* 2018) as the baseline model. DeepDTA utilizes two parallel CNN blocks to extract the biological features of drug–target pairs. It also used a simple concatenate operation to fuse the extracted features and forecast their interaction strength. Here, we introduce the new models from DrugMGR by orderly removing the multigranular representation learning, protein representation learning and reconstruction or pairwise interaction mapping blocks (see Supplementary Table S3). For the *variants-1*, the multigranular representation learning block is replaced with simple fingerprints or CNNs to learn the molecular features. The extended connectivity fingerprints (ECFPs), a class of topological fingerprints for molecular substructure characterization (Rogers and Hahn 2010), is used in variants-1. For the *variants-2*, we also use CNN or Smith–Waterman score (Wang *et al.* 2014) to represent the proteins instead of VAE module. Table 4 presents the results of the baseline model and variant models that have different elements.

**Table 4.** The average CI, MSE and $r_{m}^{2}$ values were obtained on Davis and KIBA datasets during the module ablation.^a^

Dataset	Model	Ligand	Protein	Interaction	CI	MSE	$r_{m}^{2}$
Davis	Baseline	CNN	CNN		0.878 (0.004)	0.261 (0.007)	0.630 (0.017)
	Variants-1	ECFPs	VAE	PIM	0.886 (0.005)	0.257 (0.004)	0.675 (0.013)
	Variants-1	CNN	VAE	PIM	0.898 (0.003)	0.231 (0.002)	0.713 (0.005)
	Variants-2	MGR	S-W	PIM	0.891 (0.004)	0.242 (0.004)	0.708 (0.007)
	Variants-2	MGR	CNN	PIM	0.902 (0.002)	0.224 (0.003)	0.721 (0.005)
	DrugMGR	MGR	VAE	PIM	**0.911 (0.001)**	**0.211 (0.001)**	**0.734 (0.002)**
KIBA	Baseline	CNN	CNN		0.863 (0.002)	0.194 (0.011)	0.673 (0.009)
	Variants-1	ECFPs	VAE	PIM	0.881 (0.005)	0.163 (0.006)	0.735 (0.005)
	Variants-1	CNN	VAE	PIM	0.895 (0.003)	0.139 (0.002)	0.796 (0.002)
	Variants-2	MGR	S-W	PIM	0.885 (0.004)	0.166 (0.005)	0.748 (0.004)
	Variants-2	MGR	CNN	PIM	0.897 (0.002)	0.142 (0.002)	0.793 (0.002)
	DrugMGR	MGR	VAE	PIM	**0.907 (0.002)**	**0.131 (0.001)**	**0.801 (0.002)**

a ECFPs, the extended connectivity fingerprints; S-W, Smith–Waterman score; MGR, multigranular representation learning block; PIM, pairwise interaction mapping block. Bolded: best results.

The table indicates that the performance of variants-1 (without the multigranular representation learning module) drops dramatically compared with DrugMGR. However, the introduced variants-1 consistently improves on the baseline model by 0.08, 0.018 (ECFPs) and 0.020, 0.032 (CNN) in term of CI on the Davis and KIBA datasets, respectively. The main reason for this is that the proposed block learns intricate natural mechanisms (e.g. atomic environments, chemogenomic sequences, and mutual effects), which are crucial for the interaction pattern of the ligand–protein complex. Thus, the multigranular molecular representation is very appropriate and necessary for a tailor-made black-box model. According to the performance achieved by variants-2, it can effectively fuse the feature representations and enrich the interaction information as compared with the baseline method. Specifically, variants-2 (CNN) achieves 14.2% and 26.8% performance gains in terms of *MSE* on two benchmarks, marking a significant improvement.

To further validate the effectiveness of the interaction feature extraction block (variants-3), we set three interaction manners, namely, concatenation (Öztürk *et al.* 2018), similarities-based attention (Zeng *et al.* 2021) and mix-decoder (Hua *et al.* 2023). We first replace the pairwise interaction mapping module with a simple concatenation in variants-3, resulting in 0.884 ± 0.003 (CI) and 0.245 ± 0.004 (MSE) on the Davis dataset, and 0.871 ± 0.005 (CI) and 0.181 ± 0.004 (MSE) on the KIBA dataset (see Supplementary Table S4). For a more intuitive explanation, we compare three interaction feature extractors with ours by drawing scatterplots of the ground truth affinities versus the predicted affinities for the BindingDB dataset. As shown in Supplementary Fig. S2, the model’s predicted affinities are generally smaller than the measured ones when applying concatenation or similarities-based attention. Due to the BindingDB dataset containing numerous samples with smaller label values than those with larger values. However, both the pairwise interaction mapping and mix-decoder blocks effectively mitigate the unbalanced convergence, but our proposed pairwise interaction mapping block is more competitive. This can be attributed to the possession of well-designed mechanism for modeling interaction features of ligand–protein complexes.

### 3.5 Binding regions prediction

A further strength of DrugMGR is to provide explicit interpretation and molecular insights that are critical for binding region prediction, combing the reconstructed protein and the target–ligand response vector. We compare our proposed method with existing methods for predicting the binding regions of ligand–protein pairs to demonstrate the superiority of DrugMGR. The probability of real binding sites falling into the predicted areas is adapted as the metric for judging the predictive capability of these methods. We define the binding region scale as *R* amino acid elements, and the midpoint forms the highest value in BR mentioned in Section 2.4. Table 5 evaluates the prediction results for the binding region under three scales (*R *=* *5, 10, and 15). Apparently, the accuracy of these approaches is positively correlated with the scale *R*, such that the larger scale, the better performance.

**Table 5.** Performance results of different methods in predicting the binding regions, using accuracy values.^a^

Datasets	Methods	*R* = 5	*R* = 10	*R* = 15	PDBbind
BindingDB	MGPLI	0.227	0.493	0.626	0.417
	MFR-DTA	0.334	0.579	0.681	0.536
	DrugBAN	0.528	0.632	0.749	0.619
	DrugMGR	**0.672**	**0.734**	**0.835**	**0.706**
KIBA	MGPLI	0.324	0.539	0.637	0.457
	MFR-DTA	0.451	0.602	0.742	0.492
	DrugBAN	0.547	0.662	0.783	0.568
	DrugMGR	**0.603**	**0.701**	**0.804**	**0.621**
Davis	MGPLI	0.091	0.258	0.512	0.173
	MFR-DTA	0.174	0.459	0.638	0.214
	DrugBAN	0.206	0.513	0.692	0.259
	DrugMGR	**0.354**	**0.657**	**0.733**	**0.432**

a Bolded: best results.

As shown, our proposed method consistently outperforms the baseline methods in three benchmark datasets. The BindingDB and KIBA datasets hold fewer protein types and have more interaction samples, all the models thus are easier to identify the binding region. When *R *=* *15, the accuracy performance in these two benchmarks is 0.835 and 0.804, respectively, which is superior to other models. However, the performance of all methods drops significantly in the Davis dataset when *R* <10, due to the relatively large number of protein types. Furthermore, we introduce a brand-new dataset, PDBbind (Wang *et al.* 2005), which contains structural binding site information, to examine the predictive capability of unseen protein–ligand complex’s binding regions with *R *=* *15. The results also demonstrate that the supervised learning manner for binding region prediction makes the performance more reliable.

### 3.6 Identify potential compounds for TNBC and visualization

Triple-negative breast cancer (TNBC) accounts for roughly 10% of all breast cancers, and it is known for its clinical aggressiveness, poor prognosis, and lack of effective targeted treatments (Bianchini *et al.* 2022). Poly (ADP-ribose) polymerase-1 (PARP1) is a highly conserved enzyme focused on the self-repair of cellular DNA damage. PARP1 inhibitors have been reported and used for breast cancer therapy in recent years, especially in TNBC (Mehta *et al.* 2021). Thus, we use the proposed DrugMGR in the DrugBank dataset (Knox *et al.* 2024) for the de novo identification of the potential inhibitors and chemotherapeutic agents to target the PARP1 for TNBC treatment. For the identification results, we arrange the potential compounds based on the prediction scores. The listed binding compounds are verified through the GeneCards (Stelzer *et al.* 2016) and PDB datasets. As shown in Table 6, the top 10 potential compounds for PARP1 identified by DrugMGR are presented.

**Table 6.** Top 10 identified potential compounds for target PARP1 using DrugMGR.

Rank	Compounds name	DrugBankID	Evidence (PDB ID)
1	Talazoparib	DB11760	PMID:25195882 (4PJT)
2	Olaparib	DB09074	PMID:33361107 (7KK4)
3	Amitriptyline	DB00321	PMID:28442756 (5HA9)
4	Iniparib	DB13877	Unknown
5	Nicotinamide	DB02701	Unknown
6	Niraparib	DB11793	PMID:28001384 (4R6E)
7	Theophylline	DB00277	Unknown
8	3-Methoxybenzamide	DB03073	PMID:9521710 (3PAX)
9	Rucaparib	DB12332	PMID:32241924 (6VKK)
10	Paclitaxel	DB01229	Unknown

**Figure 3.**
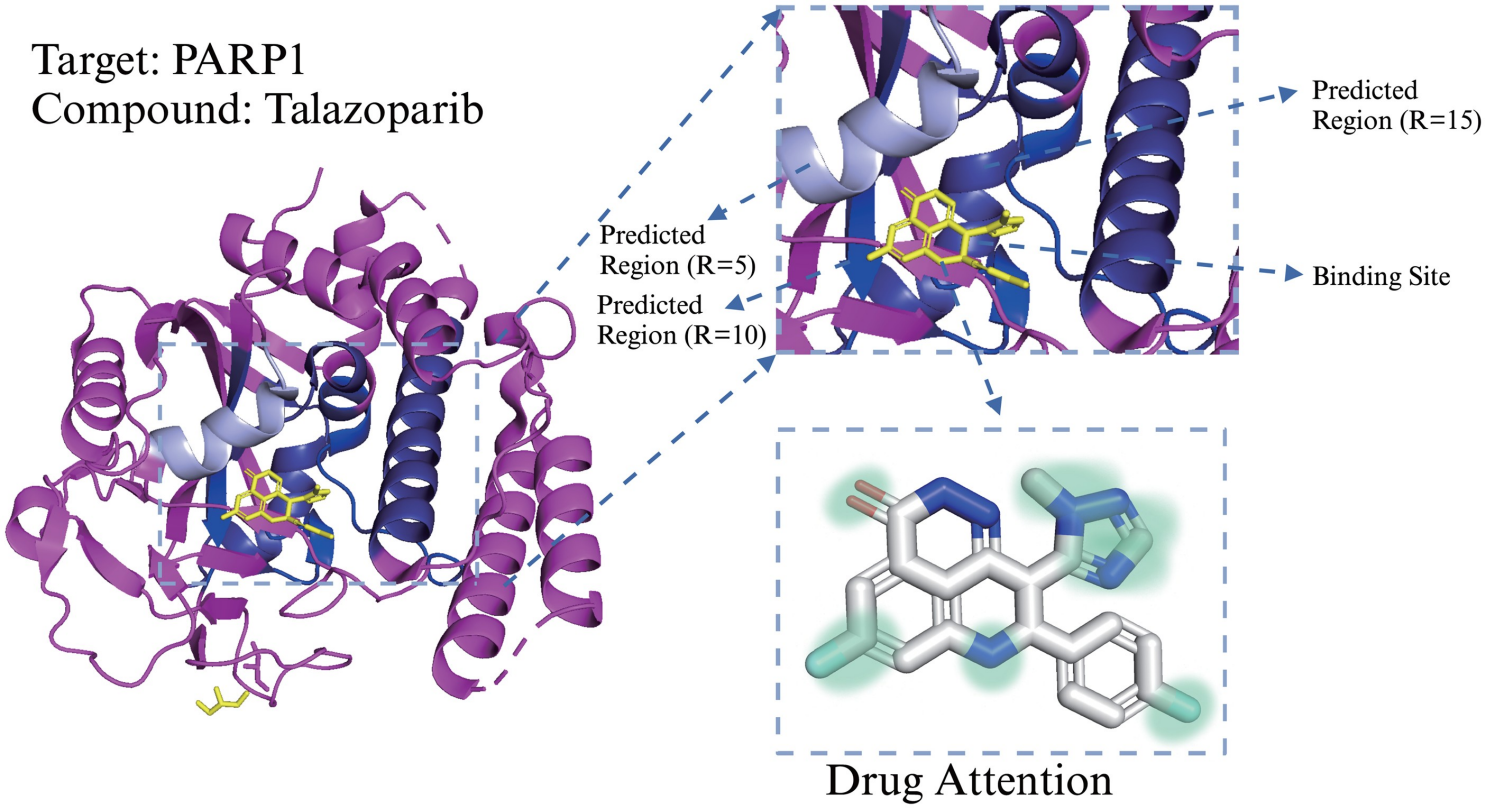
Visualization of identified drug Talazoparib and targeted PARP1 at three predicted regions, which is verified by PDB ID: 4PJT

We also visualize the binding region between PARP1 and Talazoparib (PDB ID: 4PJT) to further confirm the effectiveness of the proposed model (Aoyagi-Scharber *et al.* 2014). Figure 3 exhibits a magnified image of the practical binding site and predicted region for the ligand. Here, we represent the purple part as the noninteraction region. The light blue, blue and deep blue parts are the predicted regions with *R *=* *5, 10, and 15. The yellow position is the actual binding site of PARP1. As shown, the predicted region with region *R *>* *5 accurately falls into the binding sites. We also displayed the attention scores between ligand atoms and protein binding regions (see Supplementary Fig. S3). Intuitively, the proposed DrugMGR holds outstanding performance in predicting binding regions, providing a powerful virtual screening tool for targeting PARP1. The results demonstrate that our proposed model could enlighten biomedical scientists and enable them to screen better compound combinations for cancer treatment.

## 4 Conclusion

Here, we present a novel bioactive molecule binding prediction method called DrugMGR with multigranular representation learning and protein construction to assist in screening the potential compounds that target proteins for cancer research. We first extract the intricate molecular mechanisms (i.e. atomic environments, chemogenomic sequence, and mutual effects) with the use of multigranular feature representations for ligands. Then, we learn the high-level features of proteins through a tailor-made variational autoencoder, reconstructing the protein to highlight the binding sites. Following this, we apply pairwise representation learning of ligand–target pairs to simultaneously enrich comprehensive affinity features and predict binding regions. Finally, we predict the binding affinity of bioactive molecules by applying fully connected networks. Intriguingly, through an analysis of the effectiveness of the proposed model, we compare it with other cutting-edge methods and notice that DrugMGR significantly outperforms the SoTA methods on three benchmark datasets under different experimental strategies. Furthermore, by visualizing the positional relationships of PARP1 and corresponding compounds, our model can provide biological insight for interpreting the natural interaction of TNBC-related targets and their active compounds.

## Supplementary Material

btae176_Supplementary_Data

## Data Availability

All the data source and code in this article are available at https://github.com/lixiaokun2020/DrugMGR
