# Supplementary material for: DrugMGR: a deep bioactive molecule binding method to identify compounds targeting proteins
Source: Bioinformatics. 2024 Apr 1;40(4):btae176. doi: 10.1093/bioinformatics/btae176 (PMC11015954; doi:10.1093/bioinformatics/btae176)
Supplement: btae176_Supplementary_Data [file btae176_supplementary_data.pdf]

Supplementary Information

DrugMGR: a deep bioactive molecule binding method to identify compounds targeting proteins

Xiaokun Li[1,3,9,10], Qiang Yang[2], Long Xu[1], Weihe Dong[5], Gongning Luo[8], Wei Wang[4,*], Suyu Dong[5,*], Kuanquan Wang[1], Ping Xuan[7] , Xianyu Zhang[6] and Xin Gao[8,*]

[1]School of Computer Science and Technology, Harbin Institute of Technology, West Dazhi Street, 150001, Harbin, China.
[2]School of Medicine and Health, Harbin Institute of Technology, Yikuang Street, 150000, Harbin, China.
[3]School of Computer Science and Technology, Heilongjiang University, Xuefu Road, Harbin 150080, China.
[4]School of Computer Science and Technology, Harbin Institute of Technology, 518055, Shenzhen, China.
[5]College of Computer and Control Engineering, Northeast Forestry University, Hexing Road, 150040, Harbin, China.
[6]Department of Breast Surgery, Harbin Medical University Cancer Hospital, Haping Road, Harbin 150081, China.
[7]Department of Computer Science, School of Engineering, Shantou University, Daxue Road, 515063, Shantou, China.
[8]Computer, Electrical and Mathematical Sciences & Engineering Division, King Abdullah University of Science and Technology, 4700 KAUST, Thuwal 23955, Saudi Arabia.
[9]Postdoctoral Program of Heilongjiang Hengxun Technology Co., Ltd., Xuefu Road, 150090, Harbin, China.
[10]Shandong Hengxun Technology Co., Ltd., Miaoling Road, 266100, Qingdao, China.
*Corresponding authors:
Wei wang, School of Computer Science and Technology, Harbin Institute of Technology (Shenzhen), 518055, Shenzhen, China; E-mail: wangwei2019@hit.edu.cn； Suyu Dong, College of Computer and Control Engineering, Northeast Forestry University, Hexing Road, 150040, Harbin, China; E-mail: dongsuyu@126.com; Xin Gao, Computer, Electrical and Mathematical Sciences & Engineering Division, King Abdullah University of Science and Technology, 4700 KAUST, Thuwal 23955, Saudi Arabia; E-mail: xin.gao@kaust.edu.sa.

Data source and availability

In this work, we applied three benchmark datasets to evaluate the performance of the deep multigranular drug representation model for drug-target binding affinity prediction, namely, the Davis [1], KIBA [2] and bindingDB [3]. Meanwhile, a new dataset, PDBbind v.2019 [4], was introduced to estimate the binding region prediction. Additionally, we also utilized the PubChem database [5], ChEMBL database [6], the Protein Data Bank database [7], Gene Card database [8], and DrugBank database [9] as tools to process the feature information of compounds and proteins. The target-specific compound identification task is conducted on DUD-E [10] and LIT-PCBA [11] datasets. Statistical and chemical information of the used datasets are presented in **Figure S1** and **Table S2**.

BindingDB (https://www.bindingdb.org/rwd/bind/index.jsp) is an open-source database, which covers experimental-based assays to determine the binding strength between drugs and proteins. We used the universal subset of the dataset judged by the dissociation constant K_D that contains 31,239 DTI samples among 6,704 drugs and 587 proteins. We confirmed that the interaction between a compound and its protein existed if the corresponding K_D value was < 30 units.

The **Davis** dataset comprised 68 ligands and 442 proteins for 30,056 DTI samples. It describes the binding affinities of the kinase protein cluster and the correlated inhibitors with their respective K_D values.

The **KIBA** dataset uses a KIBA binding score, which is a statistical combination of kinase inhibitor bioactivities from the K_D, inhibition constant K_I, and half-maximal inhibitory concentration (IC_{50}), to identify the interactions between drugs and targets. According to He et al. [12], we filter it to involve 2,111 unique drugs and 229 unique targets, with a ratio of 24.4% on affinity quantity density.

PubChem (http://pubchem.ncbi.nlm.nih.gov) is a public repository for biological activity data of small molecules and RNAi reagents. The PubChem BioAssay database currently contains 500,000 descriptions of assay protocols, covering 5000 protein targets, 30,000 gene targets and providing over 130 million bioactivity outcomes. In this study, we use it to collect the SMILES strings and chemical feature information of drugs.

ChEMBL (https://www.ebi.ac.uk/chembl/) is a manually curated database of bioactive molecules with drug-like properties. It brings together chemical, bioactivity and genomic data to aid the translation of genomic information into effective new drugs.

The Protein Data Bank (PDB; http://www.rcsb.org/pdb/) is the single worldwide archive of structural data of biological macromolecules. We use PDB to describe the 3D structure information of proteins, such as coordinates, covalent bond distances and angles.

GeneCards (https://www.genecards.org/) is a searchable, integrative database that provides comprehensive, user-friendly information on all annotated and predicted human genes. The knowledgebase automatically integrates gene-centric data from ~150 web sources, including genomic, transcriptomic, proteomic, genetic, clinical and functional information.

DrugBank (https://www.drugbank.ca) is a comprehensive online database that provides detailed information about drugs, including their chemical structures, pharmacological properties, mechanisms of action, indications, side effects, drug interactions, and much more.

DUD-E (http://dude.docking.org/), a directory of useful decoys, is designed to help benchmark molecular docking programs by providing challenging decoys. It contains: 1. 22,886 active compounds and their affinities against 102 targets, an average of 224 ligands per target; 2. 50 decoys for each active having similar physico-chemical properties but dissimilar 2-D topology.

LIT-PCBA (https://drugdesign.unistra.fr/LIT-PCBA/) is a public dataset for machine learning-based compound activity prediction. It contains structural information on many small molecule compounds and their activity data in hundreds of bioactivity evaluation systems.

We claim that all the data used in this research are from public resources and there are no interest conflicts.

Evaluation metrics

In this study, we applied the *CI, MSE,* r_m^2, and *Area under the Precision-Recall Curve* (AUPR) metrics to evaluate the proposed DrugMGR. *CI* is a metric to reflect the correctness of forecasting the order of interaction strengths, and it is calculated as:

$$CI = \frac{1}{Z} \sum_{\delta_x > \delta_y} \phi(b_x - b_y)$$

where b_x and b_y are the prediction values corresponding to the label values δ_x and δ_y, respectively. Z is a normalization constant, and $\phi(x)$ is the piecewise function:

$$\phi(x) = \begin{cases} 0, if\ x < 0 \\ 0.5, if\ x = 0 \\ 1,\ \ ifx > 0 \end{cases}$$

CI measures the probability of correctly ordering the predicted and labeled affinities, and it varies from 0 to 1.

The r_m^2 index, which is also adopted in DeepDTA, can measure the external predictive performance of quantitative structure-activity relationship (QSAR) models [13]. Higher r_m^2 values of the method for the test set indicate a more promising performance. It can be calculated as follows:

$$r_m^2 = r^2 * (1 - \sqrt{r^2 - r_0^2})$$

where r^2 and r_0^2 denote the squared correlation coefficients with and without intercept, respectively.

AUPR is a metric to evaluate the model performance trained on an unbalanced dataset. Generally, AUPR is regarded as the area under the *Precision-Recall curve*, where *Precision* is the Y-axis and *Recall* is the X-axis.

$$Precision = \frac{TP}{TP + FP}, Recall = \frac{TP}{TP + FN}$$

where TP indicates the true positive samples, FP indicates the false positive samples and FN is the false negative samples.

Baseline

In this paper, we compared DrugMGR with four cutting-edge prediction methods to evaluate the performance of binding affinity predictions, namely, DeepDTA [14], GraphDTA [15], MFR-DTA [16], and DrugBAN [17]. Simultaneously, we compare our proposed method with MGPIL [18], MFR-DTA, and DrugBAN for binding region predictions. In the target-specific compound identification task, MONN [19], MFR-DTA and IGT [20] are compared.

DeepDTA employs three-layers CNNs as Protein Encoder and Compound Encoder to encode the protein sequences and the compound SMILES strings, respectively. Then, for the Interaction Estimator, the encoded protein and compound are concatenated to predict the affinity score.

GraphDTA regards each compound as a graph and attempts several GNNs, such as GIN, GAT, GCN and GAT-GCN, as the Compound Encoders to represent the compounds. In the meantime, GraphDTA regards each protein as a sequence and adopts CNNs as the Protein Encoder to encode the proteins. Then, the feature representations of drugs and proteins are combined and fed into two fully connected layers to estimate the interaction score.

MFR-DTA designs a Multi-Functional and Robust Drug–Target binding Affinity prediction model, which has three main components, i.e. biological sequence feature extraction block, Elem-feature fusion block and Mix-Decoder block. It can predict the drug-target binding affinity and binding regions simultaneously. The drug and target features are extracted by convolutional neural networks and Multilayer Perceptron. Then, the extracted features are refined by the attention mechanism and element addition. Finally, the refined feature representations are decoded by self-enhancement and cross-enhancement to predict the desired affinities and regions.

DrugBAN is an interpretable bilinear attention network with domain adaptation for drug-target interaction prediction. Given an input drug-target pair, DrugBAN first uses separate GCN and 1D convolutional neural network (1D CNN) blocks to encode molecular graph and protein sequence information, respectively. Then, it employs a bilinear attention network module to learn local interactions between encoded drug and protein representations. The bilinear attention network consists of a bilinear attention step and a bilinear pooling step to generate a joint representation. Second, a fully connected classification layer learns a predictive score, indicating the probability of interaction. To improve model generalization performance on cross-domain drug-target pairs, DrugBAN also further embeds conditional domain adversarial network into the framework to adapt representations for better-aligning source and target distributions.

MGPIL is a multigranularity protein-ligand interaction (MGPLI) model, which adopts the Transformer encoders to represent the character-level features and fragment-level features, modeling the possible interaction between residues and atoms or their segments.

MONN is a multi-objective neural network, which not only accurately predicts the binding affinities but also successfully captures the non-covalent interactions between compounds and proteins. Predictions of MONN can be validated by known chemical rules.

IGT takes as input the 3D structure of the complex after docking, and first goes through a feature extraction module to obtain graph representations of the complex, protein, and small molecule, respectively.

DrugMGR implementation

We implement the proposed model in Pytorch 1.6 under the Python 3.7 version. For optimization parameters, the Adam optimizer with learning rate 1e-4 and the weight decay 5e-3 are set. The batch size and dropout ratio are 64 and 0.1, respectively. The output block contains two fully connected layers with sizes 128 and 1. We allow the model to run at most 100 epochs for all datasets. The best-performing model with the highest Concordance Index (CI) is selected in an epoch on the validation set, and we evaluated it in terms of the test set as the final performance.

Table S1. The detailed hyperparameter settings of DrugMGR. The symbol "–" indicates the same setting as before.

Hyper-parameters	BindingDB	KIBA	Davis	PDBbind
ϑ_p	1000	1000	1200	1000
ϑ_g, ϑ_c, ϑ_t	100, 100, 50	90, 90, 50	85,85,50	100,100,50
L_g, L_c, L_t	128,128,128	-	-	-
E_g, E_c, E_t	100,100,384	-	-	-
L_p	128	-	-	-
W_{cnn}	[32,64,96]	-	-	-
W_{en}	[32,64,96]	-	-	-
W_{de}	[96,64,32]	-	-	-
ψ	[0.1-0.9]	-	-	-

Statistical and chemical information

The ligand atoms in the datasets are generally less than 100, and 90% of protein sequences in these datasets have less than 1600 of them. The number of ligands in the PDBbind dataset has a relatively positive distribution, while the numbers in the KIBA and BindingDB datasets are more centralized. Empirically, the chemical properties of ligands in Davis and KIBA are similar, including weights, topological polar surface areas, oil-water partition coefficients, and other features. However, the properties of PDBbind are quite different from other benchmarks. Thus, we evaluate the proposed model using PDBbind to identify the binding regions of unseen ligand-protein complexes. For the label distributions of the benchmarks, Davis and BindingDB are extremely unbalanced, with affinity values aggregating at around 5. The distribution of KIBA is concentrated in the central part, although it is more balanced. The distribution of the introduced PDBbind is close to a Gaussian distribution and presents a wide label range, from 2 to 12.

Table S2. Description and statistics of the benchmark datasets.

	BindingDB	KIBA	Davis	PDBbind
No.Proteins	1615	229	442	1397
No.Ligands	129,109	2,111	68	3573
No.Interaction	144,525	118,254	30,056	17,678
No.inactive interactions	41,487	22,729	2,457	-
No.non-inactive interactions	87,622	95,525	27,599	-
No.Train samples	73,018	98,525	25,046	-
No.Test samples	14,604	19,709	5,010	-

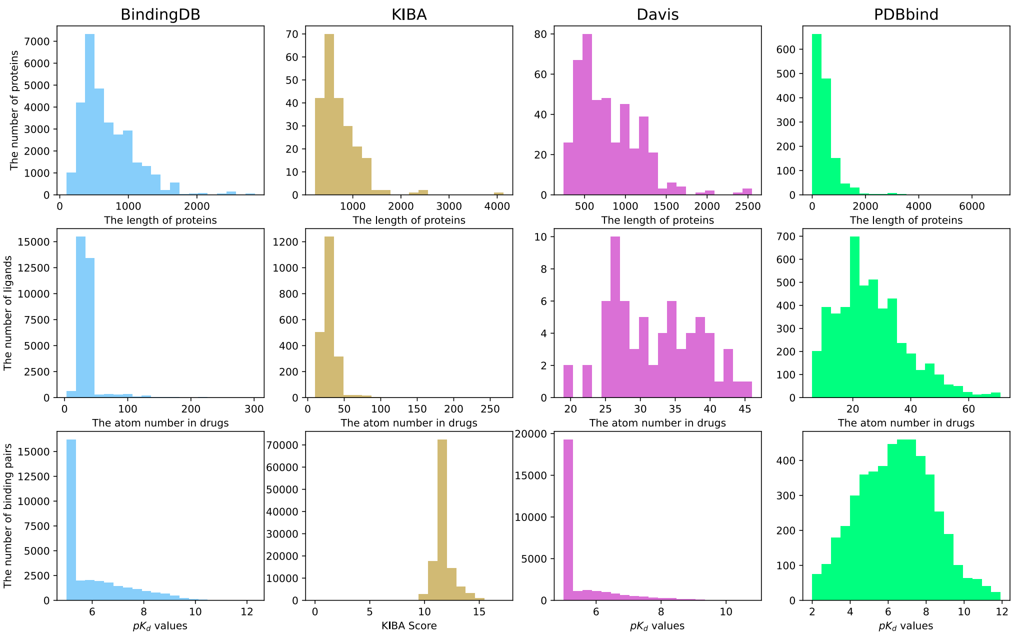

Figure S1. Chemical information of utilized datasets, i.e. BindingDB, KIBA, Davis and PDBbind datasets. The first row records the amino acid sequence length of proteins, the second row collects the atom number of ligands, as well as the third row represents the distribution of binding affinities.

Ablation study

Table S3. Detailed descriptions of the variants of DrugMGR.

Variants	Multigranular drug representation learning	Protein representation and reconstruction	Pairwise interaction mapping
Variants-1	-	✓	✓
Variants-2	✓	-	✓
Variants-3	✓	✓	-
DrugMGR	✓	✓	✓

Table S4. Results of Variants-3 on the Davis and KIBA datasets. Con: concatenation, S-A: similarity-based attention, Mix: mix decoder and PIM: pairwise interaction mapping block.

Dataset	Model	Ligand	Protein	Interaction	CI	MSE	r_m^2
Davis	*Variants-3*	MGR	VAE	Con	0.884(0.003)	0.245(0.004)	0.684(0.007)
	Variants-3	MGR	VAE	S-A	0.899(0.004)	0.231(0.004)	0.708(0.005)
	Variants-3	MGR	VAE	Mix	0.905(0.002)	0.219(0.002)	0.727(0.001)
	DrugMGR	MGR	VAE	PIM	**0.911(0.001)**	**0.211(0.001)**	**0.734(0.002)**
KIBA	*Variants-3*	MGR	VAE	Con	0.871(0.005)	0.181(0.004)	0.725(0.004)
	Variants-3	MGR	VAE	S-A	0.887(0.004)	0.157(0.002)	0.763(0.005)
	Variants-3	MGR	VAE	Mix	0.892(0.002)	0.149(0.002)	0.796(0.003)
	DrugMGR	MGR	VAE	PIM	**0.907(0.002)**	**0.131(0.001)**	**0.801(0.002)**

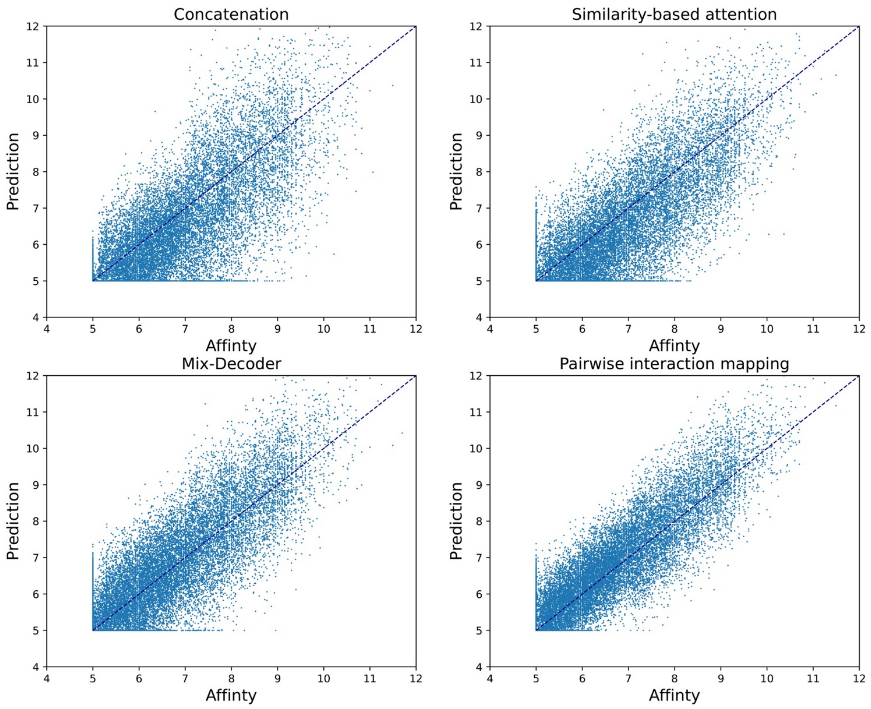

Figure S2. The performance of different interaction methods, i.e. concatenation, similarity-based attention, Mix-decoder, and pairwise interaction mapping.

Interpretation of multigranular attention

In this study, we have already demonstrated the effectiveness of our proposed method by predicting the potential inhibitors and chemotherapeutic agents with TNBC. To further interpret the interaction mechanism in the pairwise interaction mapping block, we display the attention scores of potential PAPR1 binding regions and corresponding Talazoparib atoms in each granular representation. We anchored 4 regions, and their sequences and positions are shown (see **Table S5**). **Figure S3** explicitly exhibits the contribution of each Talazoparib's intricate natural characteristics (i.e., atomic environments, chemogenomic sequence, and mutual effects) to the final binding regions of PAPR1.

Table S5. Binding Region IDs, amino acid sequences and corresponding positions of PAPR1.

Region IDs	Amino acid sequences	Positions
1	QLPGVKSEGKRKGDEVDGVDEVAKKKSKKEKDKDSK	198-233
2	GALLPCEECSGQLVFKSDAYYCTGDVTAWTKCMVKTQTPNRKE	290-332
3	TSASVAATPPPSTASAPAAVNSSAS	361-385
4	TASAPAAVNSSASADKPLSNMKILTLGKLSRNKDEVKAMIEKLGGKLT	373-524

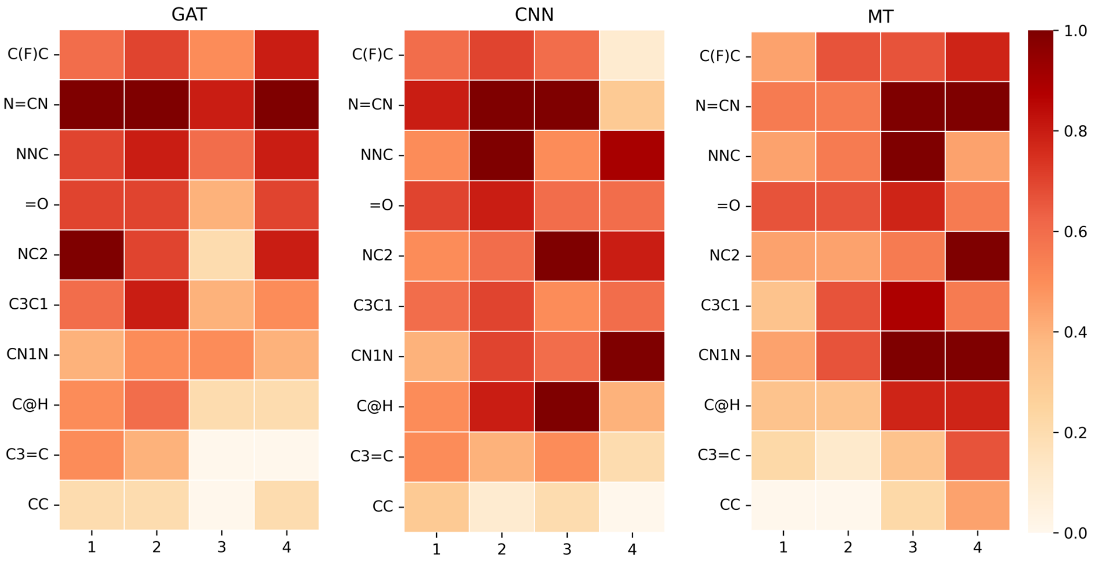

Figure S3. The attention scores between Talazoparib atoms and PAPR1 binding regions. Br is an abbreviation of the benzene ring.

Reference

[1] Davis, M.I. et al. (2011) Comprehensive analysis of kinase inhibitor selectivity. *Nat. Biotechnol.*, **29**, 1046–1051.

[2] Tang, J. et al. (2014) Making sense of large-scale kinase inhibitor bioactivity data sets: a comparative and integrative analysis. *J. Chem. Inf. Model.*, **54**, 735–743.

[3] Gilson, M. et al. (2016). BindingDB in 2015: A public database for medicinal chemistry, computational chemistry and systems pharmacology. *Nucleic acids research*, **44**(D1), D1045–D1053.

[4] Wang, R. et al. (2005) The PDBBind database: methodologies and updates. *J. Med. Chem.*, **48**, 4111–4119.

[5] Bolton,E.E. et al. (2008) Pubchem: integrated platform of small molecules and biological activities. *Annu. Rep. Comput. Chem.*, **4**, 217–241.

[6] Gaulton, A. et al. (2011). ChEMBL: a large-scale bioactivity database for drug discovery. *Nucleic acids research*, **40**(D1).

[7] Berman H. et al. (2000). The Protein Data Bank. *Nucleic Acids Res*, **28**(1), 235-242.

[8] Stelzer, G. et al. (2016). The GeneCards Suite: From Gene Data Mining to Disease Genome Sequence Analyses. *Current protocols in bioinformatics*, **54**, 1.30.1–1.30.33.

[9] Wishart, D. et al. (2018). DrugBank 5.0: a major update to the DrugBank database for 2018. *Nucleic acids research*, **46**(D1), D1074–D1082.

[10] Mysinger, M. et al. (2012). Directory of useful decoys, enhanced (DUD-E): better ligands and decoys for better benchmarking. *Journal of medicinal chemistry*, **55**(14), 6582–6594.

[11] Tran-Nguyen, V. et al. (2020). LIT-PCBA: An Unbiased Data Set for Machine Learning and Virtual Screening. *Journal of chemical information and modeling*, **60**(9), 4263–4273.

[12] He, T. et al. (2017). SimBoost: a read-across approach for predicting drug-target binding affinities using gradient boosting machines. *J Cheminform*, **9**(1), 24.

[13] Cheng, F. et al. (2012) Prediction of chemical–protein interactions: multitarget-QSAR versus computational chemogenomic methods. *Mol. Biosyst.*, **8**, 2373–2384.

[14] Öztürk, H. et al. (2018). DeepDTA: deep drug-target binding affinity prediction. *Bioinformatics*, **34**(17), i821–i829.

[15] Nguyen, T. et al. (2021). GraphDTA: predicting drug-target binding affinity with graph neural networks. *Bioinformatics*, **37**(8), 1140–1147.

[16] Hua, Y. et al. (2023). MFR-DTA: a multi-functional and robust model for predicting drug-target binding affinity and region. *Bioinformatics*, **39**(2), btad056.

[17] Bai, P. et al. (2023). Interpretable bilinear attention network with domain adaptation improves drug-target prediction. *Nat Mach Intell,* **5**(2), 126-136.

[18] Wang, J. et al. (2022). MGPLI: exploring multigranular representations for protein-ligand interaction prediction. *Bioinformatics*, **38**(21), 4859–4867.

[19] Li, S. et al. (2020). MONN: a multi-objective neural network for predicting compound-protein interactions and affinities. *Cell Systems*, **10**(4), 308-322.

[20] Liu, S. et al. (2022). Improved drug-target interaction prediction with intermolecular graph transformer. *Briefings in bioinformatics*, **23**(5), bbac162.
